# Sharp-to-Broad Band Energy Transfer in Lithium Aluminate and Gallate Phosphors for SWIR LED

**DOI:** 10.1021/acsaom.3c00464

**Published:** 2024-03-01

**Authors:** Yi-Ting Tsai, Pei-Xuan Chen, Mikołaj Kamiński, Natalia Majewska, Sebastian Mahlik, Mu-Huai Fang

**Affiliations:** † Research Center for Applied Sciences, Academia Sinica, Taipei 11529, Taiwan; ‡ Institute of Organic and Polymeric Materials, National Taipei University of Technology, Taipei 10608, Taiwan; § Institute of Experimental Physics, Faculty of Mathematics, Physics and Informatics, University of Gdansk, Wita Stwosza 57, 80-308 Gdansk, Poland; ∥ International Centre for Theory of Quantum Technologies (ICTQT), University of Gdansk, 80-308 Gdansk, Poland

**Keywords:** Short-wave infrared, Light-emitting diodes, Energy transfer, Phosphor, Transition metal ions

## Abstract

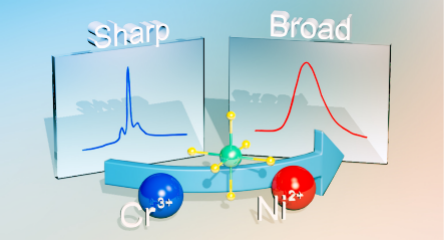

Short-wave infrared (SWIR) phosphor-converted light-emitting diode (LED) technology holds promise for advancing broadband light sources. Despite the potential, limited research has delved into the energy transfer mechanism from sharp-line to broadband emission in SWIR phosphors, which remains underexplored. Herein, we demonstrate bright SWIR phosphors achieved through Cr^3+^/Ni^2+^ energy transfer in LiGa_5(1–*x*)_Al_5*x*_O_8_. High-resolution X-ray diffraction revealed the typical solid solution and distortion occurring in Al^3+^ octahedral sites. In addition, the X-ray absorption spectrum illustrates that Cr^3+^ and Ni^2+^ have different coordination environments, showing the possibility that they occupy different positions or that the coordinated environment of Ni^2+^ is distorted due to charge imbalance. Temperature-dependent studies provide insights into the energy transfer dynamics between Cr^3+^/Ni^2+^, from the ^2^E level of Cr^3+^ (sharp band) to the ^3^T_1_ level of Ni^2+^ (broadband). The increased emission intensity at lower temperatures in the *x* = 0.6 and *x* = 1.0 samples can be explained by the positioning of the ^3^T_1_ level above the ^2^E level of Cr^3+^ ions. Finally, we established a mechanism involving a sharp line to broadband energy transfer showcasing a high-power SWIR LED with a radiant power of 21.45 mW.

## 1 Introduction

Near-infrared (NIR) phosphors play a pivotal role in modern technology, with applications extending from advanced bioimaging optical communications to security features.^1^ NIR light, with wavelengths typically between 700 and 1000 nm, is advantageous due to its low absorption in biological tissues, resulting in reduced scattering and higher penetration depths, which is crucial for non-invasive medical imaging.^2−4^ Moreover, the NIR spectral region is also critical for telecommunications, as it coincides with the low-loss window of optical fibers.^5^ In recent years, short wavelength study has become popular because of its high penetration power and characteristic absorption signal. Specifically, those capable of emitting light in the short wavelength infrared (SWIR) range of 1000–1700 nm are increasingly essential for advanced technological applications.^6−8^ This spectral region, often called the “eye-safe” region, is also crucial for light detection and ranging (LiDAR) applications where safety considerations for incidental human exposure are vital.^9−11^ In biomedical diagnostics, emissions beyond 1000 nm allow for deeper tissue penetration with reduced scattering, enhancing the capabilities of non-invasive optical imaging and improving diagnostic accuracy.^12−15^

Among various materials, lithium gallate (LiGa_5_O_8_) has emerged as a promising NIR phosphor host due to its stability and suitable optical properties.^16^ The spinel-like structured and crystalline matrix of LiGa_5_O_8_ can be doped with various rare earth and transition metal ions, which impart the desired NIR luminescence upon excitation. These doped phosphors exhibit sharp emission lines characteristic of the *f*–*f* or *d*–*d* transitions of the activator ions in six-coordination sites such as Cr^3+^, Ni^2+^, Tm^3+^, and Pr^3+^.^17−20^ Recent literature highlights the occurrence of energy transfer between Cr^3+^ (broadband emission) and Ni^2+^ (broadband emission).^21^ However, the mechanism underlying the energy transfer from sharp-band to broadband emission remains inadequately addressed.

Herein, we synthesized a series of LiGa_5(1–*x*)_Al_5*x*_O_8_:0.05Cr^3+^,0.015Ni^2+^ (LGAOCN) samples with *x* = 0.0–1.0.^22^ The distribution of Cr^3+^ and Ni^2+^ ions to different locations in the material was characterized using X-ray absorption spectroscopy (XAS). Additionally, the study delves into temperature-dependent decay time and photoluminescent characteristics to elucidate the mechanisms governing energy transfer between Cr^3+^ and Ni^2+^ ions. Lastly, we present the development of SWIR light-emitting diode (LED) devices and propose their potential applications in bioimaging in the SWIR region.^23^

## 2 Results and Discussion

**Figure 1:**
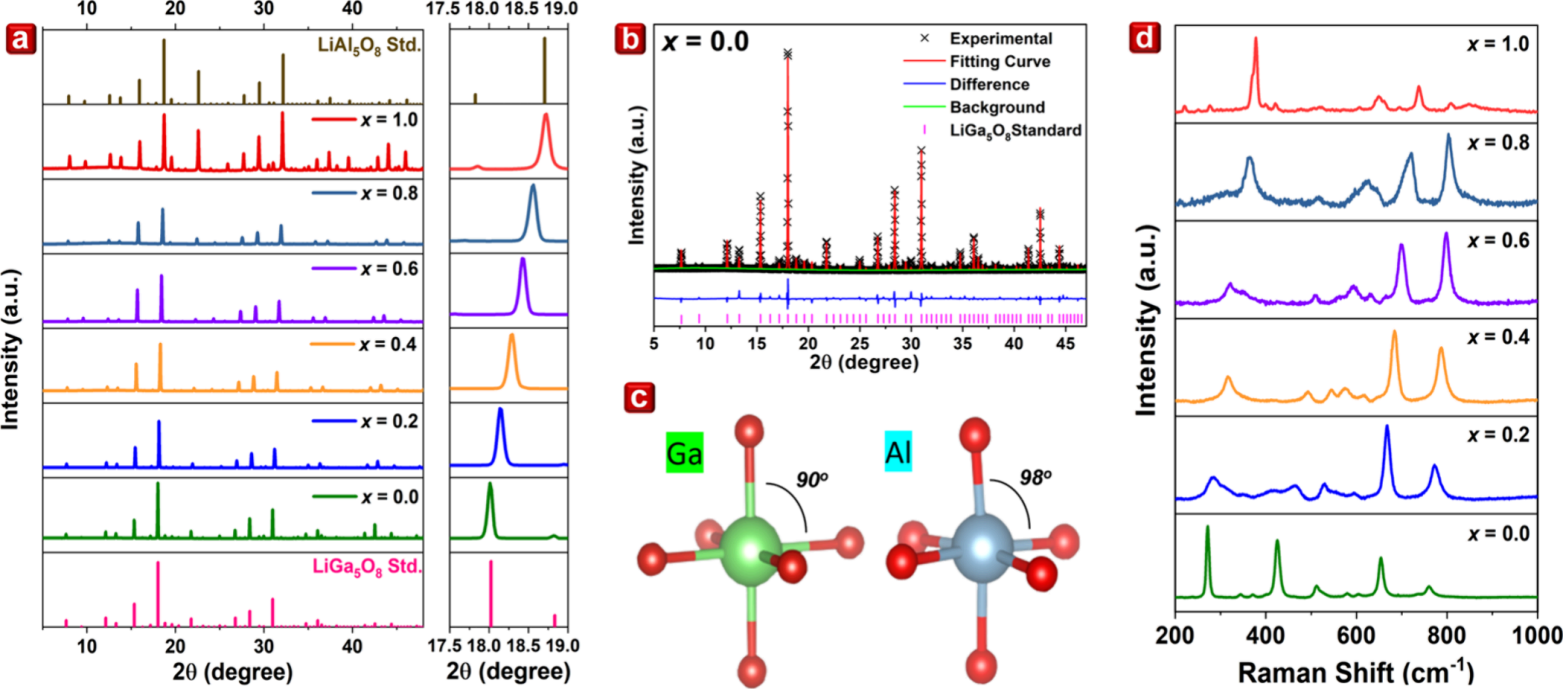
(a) XRD results of LiGa_5(1–*x*)_Al_5*x*_O_8_:0.05Cr^3+^,0.015Ni^2+^ (*x* = 0.0–1.0). (b) Rietveld refinement of *x* = 0.0 sample. (c) Scheme of GaO_6_ and AlO_6_ octahedra in LiGa_5_O_8_ and LiAl_5_O_8._ (d) Raman spectra of LiGa_5(1–*x*)_Al_5*x*_O_8_:0.05Cr^3+^,0.015Ni^2+^ (*x* = 0.0–1.0).

The LGAOCN samples with *x* = 0.0–1.0 were synthesized through a solid-state reaction. High-resolution XRD analysis revealed the existence of pure-phase solid solutions (*x* = 0.0–1.0) containing Al^3+^ doping, as shown in Figure 1a. Notably, within the 17.5° to 19.0° range, diffraction peaks exhibited a discernible shift to higher angles with increasing *x*. This phenomenon is attributed to the smaller radius of Al^3+^ ions (0.535 Å; CN = 6, CN—coordination number) compared to Ga^3+^ ions (0.62 Å, CN = 6), inducing crystal contraction.^24^ To obtain more precise structural information, Rietveld refinement was performed on LGAOCN samples with *x* = 0.0–1.0, as depicted in Figure 1b and Supporting Information Figure S1. The resultant atomic positions, occupancies, and lattice parameters are detailed in Tables S1 and S2. Remarkably, Al^3+^ ions favor occupying Ga1 sites (octahedral coordination) over Ga2 sites (tetrahedral coordination) as Al^3+^ concentration increased from *x* = 0.0 to 0.8. The inherent spinel-like structure of LiGa_5_O_8_, crystallizing in a cubic structure with *P*4_3_3_2_ space group, involves Li^+^ ions forming octahedra through corner-sharing with GaO_4_ tetrahedra and edges with other GaO_6_ octahedra. From *x* = 0.8 to 1.0, the doping with Al^3+^ induced local contraction and distortion, altering the O–Ga–O and O–Al–O angles to 90° (*x* = 0.0) and 98° (*x* = 1.0), respectively. Consequently, this led to the division of the equivalent bonding distance (2.05 Å) in LiGa_5_O_8_, in contrast with the three distinct bond lengths defined at 1.85, 1.91, and 1.94 Å in LiAl_5_O_8_.

To establish the correlation between active phonon modes and structural symmetry after Al^3+^ doping, Raman spectra analyses of LGAOCN are illustrated in Figure 1d. A noticeable trend is observed as the peaks shift toward higher wavenumbers with the incorporation of Al^3+^ ions, which is attributed to substituting Ga^3+^ ions (69.72 amu) with the lighter Al^3+^ ions (26.98 amu).^25^ In the *x* = 0.0 sample, the peak at 270 cm^–1^ is associated with the translational motion of the [GaO_4_] tetrahedra site. Additionally, peaks within the 300–600 cm^–1^ range represent the bending mode (deformation) of both the [GaO_4_] tetrahedra site and [GaO_6_] octahedra site. The broadening of the peaks from 200 to 600 cm^–1^ is ascribed to the susceptibility of translation and bending modes to external influences from other atoms at shared corners, resulting in a more disordered distribution involving Li^+^, Al^3+^, and Ga^3+^.^26^ From the *x* = 0.0 to 0.2 samples, the low Raman shift range is associated with the translational motion of tetrahedral sites, which is sensitive to neighboring atoms. When Al-doped, the Raman activity decreases due to the disorder introduced by Al and Ga. Consequently, both the intensity and symmetry in the low Raman shift range decrease. In the *x* = 1.0 end-member sample, the bending mode signals narrow. However, the stretching mode exhibits a distinctive displacement and gradual reduction in intensity. This reduction in intensity is attributed to the increasing distortion of the octahedral sites induced by the incorporation of Al^3+^, leading to a decrease in symmetry and a diminished Raman-active characteristic.^22^

During the LED manufacturing process, the aggregation and morphology of phosphor powders have an impact on their application. Therefore, microscopy technologies are important for phosphor development. Optical microscopy (OM) and scanning electron microscopy (SEM) are suitable for observing the morphology and distribution of phosphor powder. Under the OM graph, the color of the prepared powder transitions from light green to pink with increasing Al concentration, as depicted in Figure S2. Based on the OM measurement, it can be observed that the aggregation of powders is similar in all samples. On the contrary, SEM image is more suitable for measuring the morphology of phosphor. SEM imaging reveals a morphological transformation from columnar-like structures to fine particles, with the average particle size decreasing from around 5 μm to less than 500 nm, as shown in Figure S3. Given the burgeoning interest in mini-LED on wearable devices, finding suitable luminescent materials has become imperative. Generally, mini-LED chips have a size of about 5 × 9 mil, and phosphors with small particle sizes are easier to use in mini-LED devices. From SEM results, Al doping will help reduce size and apply to mini-LED.

**Figure 2:**
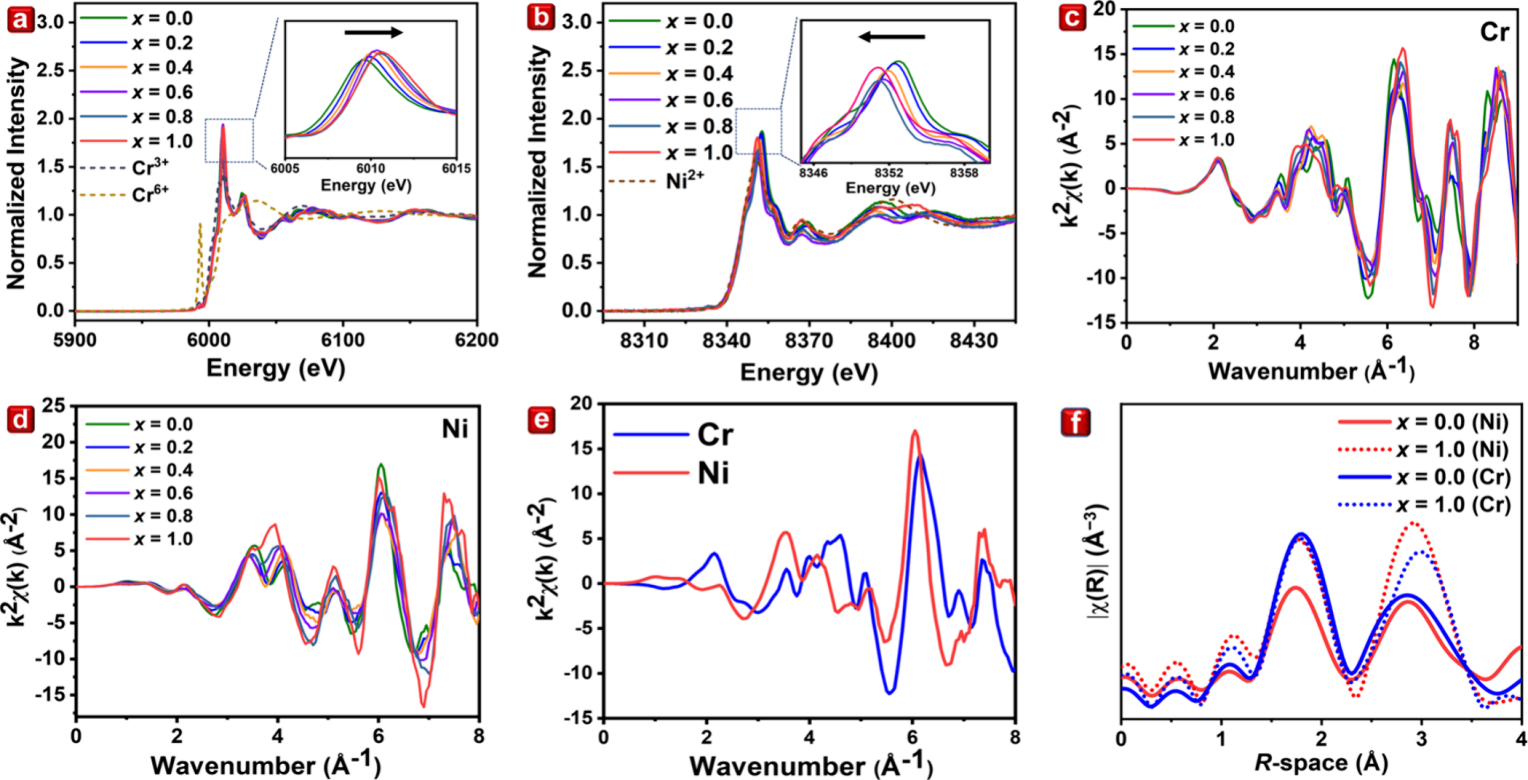
LiGa_5(1–*x*)_Al_5*x*_O_8_:0.05Cr^3+^,0.015Ni^2+^ XANES spectra with *x* = 0.0–1.0 of (a) Cr *K*-edge with Cr^3+/6+^ standard pattern and (b) Ni *K*-edge with Ni^2+^ standard pattern. (c) Cr *K*-edge *k*^2^-weighted Fourier transforms and (d) Ni *K*-edge *k*^2^-weighted Fourier transforms of EXAFS spectra. (e) Cr and Ni *K*-edge *k*^2^-weighted Fourier transforms of LiGa_5(1–*x*)_Al_5*x*_O_8_:0.05Cr^3+^,0.015Ni^2+^ with *x* = 0.0. (f) Cr and Ni *K*-edge *R*-space EXAFS spectra of LiGa_5(1–*x*)_Al_5*x*_O_8_:0.05Cr^3+^,0.015Ni^2+^ with *x* = 0.0 and 1.0.

The Cr *K*-edge and Ni *K*-edge X-ray absorption near-edge structure (XANES), which utilize synchrotron radiation, stand as formidable tools in the investigation of charge variations in materials research. As illustrated in Figure 2a, the Cr *K*-edge XANES spectra closely resemble the Cr^3+^ standard across all samples, confirming the ionic charge of chromium.^27^ Notably, the inset in the figure highlights a shift in the absorption peaks of Cr toward higher energy levels, approximately 1 eV. The increase in the Al concentration within the structure triggers a change in the oxidation states of Cr due to structural contraction, resulting in a stronger ionic Cr–O bonding. Consequently, the charge distribution shifts toward the O ion, leading to decreased electron density for Cr^3+^ ions and a reduced effective shielding of the nuclear charge. On the other hand, Figure 2b displays the Ni *K*-edge XANES spectra, with the inset demonstrating an absorption peak shift for Ni, moving nearly 1.5 eV toward low energy levels.

Furthermore, an in-depth understanding of the coordinated environment of Cr^3+^ and Ni^2+^ in the LGAOCN structure necessitates an analysis of the extended X-ray absorption fine structure (EXAFS) information. Figure 2c presents Cr *K*-edge *k*^2^-weighted Fourier transforms of EXAFS. The oscillation pattern observed in the Cr *K*-edge for samples with the *x* = 0.0–0.6 range indicates that Cr occupies the same sites. However, a subtle shift in the oscillation pattern is evident for *x* = 0.8–1.0 compared to those with *x* = 0.0–0.6 range. These results corroborate a significant change in the local environment of Cr^3+^ in samples with *x* = 0.8–1.0, aligning with earlier-discussed XRD data. Similar results could be observed in the Ni *K*-edge *k*^*2*^-weighted Fourier transforms of EXAFS (Figure 2d). Simultaneously, we compared the Cr and Ni *K*-edge *k*^*2*^-weighted Fourier transforms in Figure 2e. The two different signals of *k*^*2*^-weighted Fourier transforms reveal that the coordinated environment of Cr^3+^ and Ni^2+^ are not precisely the same. Possible reasons include Ni^2+^ occupying the Ga2 sites (GaO_4_) or Li sites (LiO_6_). Or, the Ni^2+^ ion still occupies the Ga1 sites (GaO_6_), while the charge imbalance results in a distorted coordinated Ni^2+^ environment, leading to different oscillation patterns. To elucidate the different coordinated shells of Cr^3+^ and Ni^2+^, we analyzed the *R*-space of EXAFS, as illustrated in Figure 2f. For Cr^3+^ ions, the first coordinated shell slightly shrinks with the incorporation of Al^3+^ ions. Surprisingly, the first coordinated shell of Ni^2+^ ions slightly expands by incorporating Al^3+^ ions. This may explain the opposite energy-shifting phenomenon in Figures 2a and 2b. Furthermore, this also reveals the unique advantage of the X-ray absorption techniques compared to the X-ray diffraction techniques.

**Figure 3:**
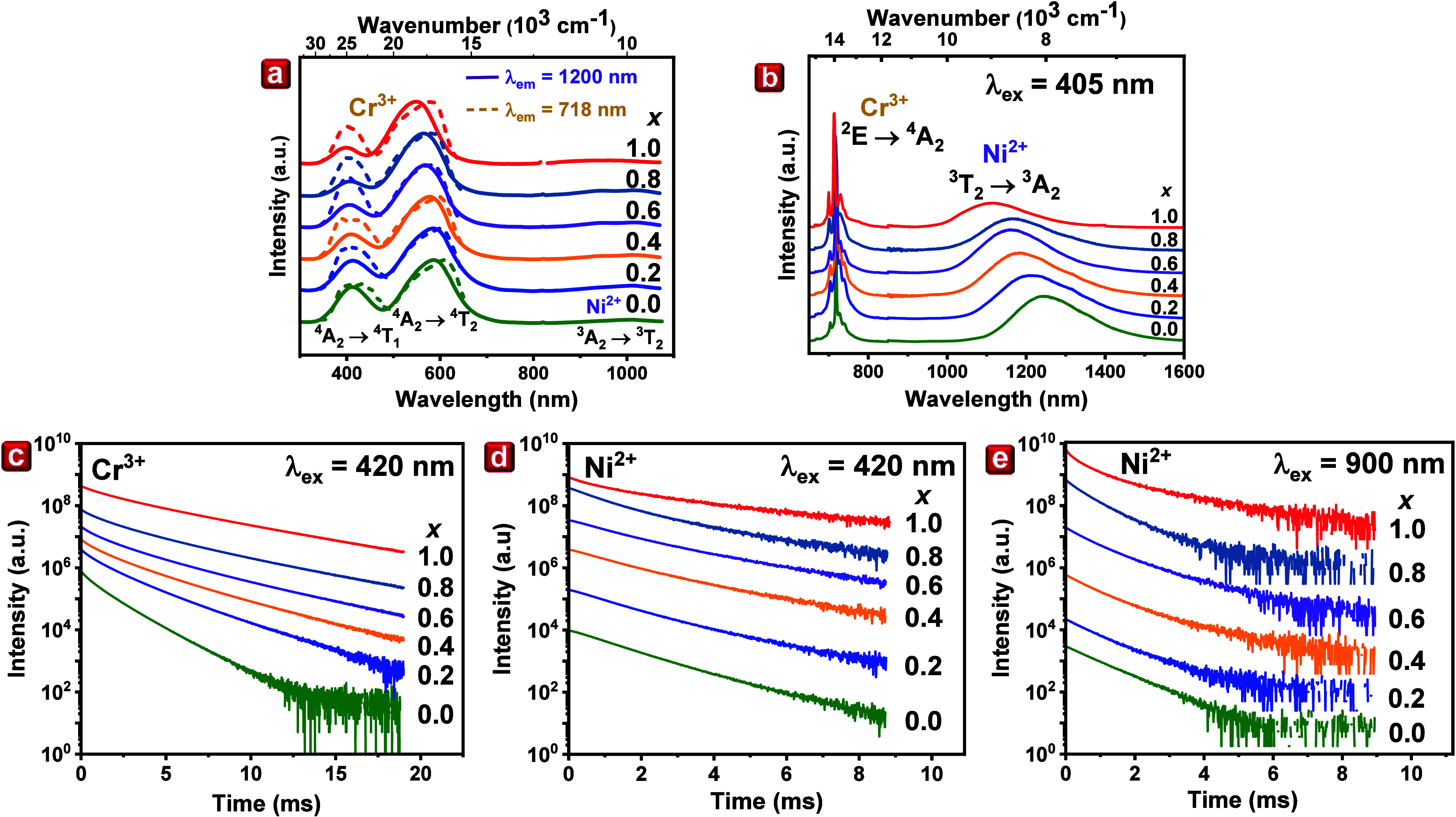
Room-temperature (a) photoluminescence excitation upon observation at 718 nm (Cr^3+^ observation, dashed line) and 1200 nm (Ni^2+^ observation, solid line) and (b) photoluminescence of Cr^3+^ and Ni^2+^ emission upon excitation at 405 nm of the LiGa_5(1–*x*)_Al_5*x*_O_8_:Cr^3+^, Ni^2+^ with *x* = 0.0–1.0. Decay profiles of (c) Cr^3+^ emission upon excitation at 420 nm, (d) Ni^2+^ emission upon excitation at 420 nm, and (e) 900 nm (direct Ni^2+^ excitation of LiGa_5(1–*x*)_Al_5*x*_O_8_:Cr^3+^, Ni^2+^ with *x* = 0.0–1.0).

To investigate the optical properties and luminescence characteristics of LGAOCN with *x* values ranging from 0.0 to 1.0, we performed photoluminescence excitation (PLE), photoluminescence (PL), temperature-dependent photoluminescence (TDPL), and photoluminescence decay profiles measurements. Figure 3a showcases the room-temperature PLE spectra of LGAOCN for *x* values ranging from 0.0 to 1.0. During observation at 718 nm for Cr^3+^, the PLE spectra exhibited two distinctive excitation bands. The excitation bands are identified as a high-energy band peaking at 410 nm (^4^A_2_ → ^4^T_1_ transition) and a lower energy band peaking at 580 nm (^4^A_2_ → ^4^T_2_ transition). Additionally, when PLE is observed at 1200 nm for Ni^2+^, two visible range excitation bands corresponding to Cr^3+^ ion excitation are observed, indicating energy transfer from Cr^3+^ to Ni^2+^. An anticipated Ni^2+^ excitation bands corresponding to the ^3^A_2_ → ^3^T_1_(^3^P) and ^3^A_2_ → ^3^T_1_ (^3^F) transition overlap with the ^4^A_2_ → ^4^T_1_ and ^4^A_2_ → ^4^T_2_ transition of Cr^3+^, respectively, resulting in an intensified excitation bands at around 400 and 600 nm.^20^ The third excitation band, notably less intense in the near-infrared range (800–1070 nm), is attributable to the ^3^A_2_ → ^3^T_2_ transition of Ni^2+^.

Interestingly, a blue shift in the ^4^A_2_ → ^4^T_1_ and ^4^A_2_ → ^4^T_2_ excitation bands of Cr^3+^, alongside a red shift in the ^2^E → ^4^A_2_ emission line of the Cr^3+^ ion, are observed. A similar blue shift is noted in the ^3^T_2_ → ^3^A_2_ emission and ^3^A_2_ → ^3^T_2_ excitation spectra of Ni^2+^ with increasing *x* values, as shown in Figure S4. These spectral shifts align with crystal field theory. According to the crystal field theory, the observed blue shift can be rationalized by the smaller Al^3+^ ions (0.535 Å, CN = 6) occupying the Ga1 octahedrally coordinated sites originally occupied by Ga^3+^ ion (0.62 Å, CN = 6). This causes the crystal lattice to shrink and the crystal field strength (*Dq*) around the Cr^3+^ and Ni^2+^ ions to increase, thereby increasing the energy transitions of ^4^T_1_ → ^4^A_2_ (Cr^3+^), ^4^T_2_ → ^4^A_2_ (Cr^3+^) and ^3^T_2_ → ^3^A_2_ (Ni^2+^). The crystal field minimally influences the energy difference between the lowest doublet ^2^E and the ground state ^4^A_2_ of Cr^3+^ emission, where the energy of this state should slightly increase (blue shift). The red shift in the ^2^E → ^4^A_2_ luminescence of Cr^3+^ from *x* = 0.0 to 0.6 suggests a predominant contribution from the nephelauxetic effect.^28^

The PL spectra, depicted in Figure 3b, delineate the emission properties of the studied samples. Under 405 nm excitation, Cr^3+^ exhibits a characteristic emission spectrum in a strong crystal field site, with narrow lines between 700 and 750 nm corresponding to the ^2^E → ^4^A_2_ spin-forbidden transition. Concurrently, a broadband emission in the SWIR range of 1000–1600 aligns with the ^3^T_2_ → ^3^A_2_ transition of Ni^2+^ ions in octahedral coordination (d^8^ configuration). The emission of Ni^2+^ undergoes a shift from 7961 cm^–1^ (*x* = 0.0) to 8897 cm^–1^ (*x* = 1.0). Figure S5a illustrates the full width at half-maximum (fwhm) values and the positions of the ^3^T_2_ → ^3^A_2_ emission of Ni^2+^. For the *x* = 0.0 sample, the fwhm is measured at 1393 cm^–1^. A significant increase in fwhm is observed for the *x* = 0.2 sample, which remains relatively stable up to *x* = 1.0. The observed increase in fwhm in Al^3+^-containing samples is attributed to an inhomogeneous distribution of Al ions, altering the crystal field surrounding Cr^3+^ ions. Notably, a discontinuity in the broadband emission shift is observed for the *x* = 0.8 sample, suggesting structural changes marked by the orange scatters in Figure S5a. Considering the trend of changing the position of the maximum of the emission band, one could expect a further blue shift of the Ni^2+^ band as *x* increases. However, between *x* = 0.6 and *x* = 0.8, there is a phase transition, which results in a step change in the position of the emission band. We also observe a similar effect for Cr^3+^ emissions. As *x* increases, there is a red shift, while for the *x* = 0.8 sample, we have a step change and a blue shift. Figure S5b presents the Ni^2+^/Cr^3+^ ratio, revealing a decrease in Ni^2+^ intensity relative to Cr^3+^ emission with increasing Al^3+^ concentration. As shown in Figure S6a, the overlapping of Cr^3+^ emission spectra and Ni^2+^ excitation spectra is stronger for the *x* = 0 sample than for the *x* = 1.0 sample, making the energy transfer more prominent for the *x* = 0 sample. This trend results from diminished energy transfer from Cr^3+^ to Ni^2+^ due to the blue shift of the Ni^2+^ excitation band (^3^A_2_ → ^3^T_1_(^3^F)), which increases the energy difference between the ^3^T_1_(^3^F) state of Ni^2+^ and the ^2^E state of Cr^3+^. Figure 3c displays room-temperature (RT) decay profiles of luminescence in samples with varying *x* values, observed at the maximum of Cr^3+^ luminescence upon 420 nm excitation. The decay profile of Cr^3+^ emission is multiexponential across all samples and increases with rising *x* values. This elongation of decay time clearly confirms a decrease in energy transfer efficiency as *x* increases. Panels d and e of Figure 3 display the decay profiles of Ni^2+^ emission under two distinct excitation wavelengths at 420 nm (also excites Cr^3+^) and 900 nm (only excites Ni^2+^), respectively.^29^ Notably, the decay profiles of Ni^2+^ luminescence upon 900 nm excitation are shorter than those measured at 420 nm. This variation suggests that the Cr^3+^ → Ni^2+^ energy transfer causes an elongation of the Ni^2+^ luminescence decay time. Where *x* increases, according to structural information, part of the Al^3+^ occupies the Li^+^ sites and vice versa. This hints that Ni^2+^ may also occupy both sites, giving rise to multiexponential decay. The average decay times for Cr^3+^ and Ni^2+^ emission were calculated using eq 1:
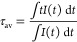
1where *I*(*t*) is emission intensity at time *t*. The calculated average decay times of Cr^3+^ emission and Ni^2+^ emission upon direct excitation are presented in Figure S5c. The average decay time for both emissions increases with increasing *x* concentration. The decay time of Cr^3+^ emission changes from 1.22 to 2.87 ms, while Ni^2+^ varies slightly from 0.91 to 1.28 ms. The different coordination environments may lead to different distances between Cr^3+^ and Ni^2+^, the shorter distance being a factor that promotes energy transfer. However, the energy transfer is decreased after doping the Al^3+^. This result indicates that the overlapping of emission (Cr^3+^) and excitation (Ni^2+^) spectra is the dominant effect for energy transfer in this case. Besides, sharp lines exhibit high intensity and long decay times. This leads to a higher probability of electrons being in the excited state and undergoing energy transfer. As a result, there is a reduction in the direct radiative recombination process. Additionally, sharp line emission is more likely to overlap the entire excitation range of Ni^2+^.

To further support the occurrence of the energy transfer, the PLE spectra of LGAO for *x* = 0.0 and *x* = 1.0 doped with only Ni^2+^ are presented in Figure S7. When observed at Ni^2+^ emission, the PLE of samples solely doped with Ni^2+^ consist of three transitions from ^3^A_2_(^3^F) to ^3^T_1_(^3^P), ^3^T_1_(^3^F), and ^3^T_2_(^3^F). When comparing these spectra to ones additionally doped with Cr (LGAOCN), the PLE bands associated with Cr^3+^ strongly dominate the whole spectrum, while the Ni^2+^ bands are not observable, demonstrating the significant impact of Cr^3+^ on Ni^2+^ luminescence. The same trend is evident in diffuse reflectance measurements (circles in Figure S7), where the distinctive features of the reflectance spectra of LGAO doped solely with Ni^2+^ are not apparent in the corresponding LGAOCN spectra.

**Figure 4:**
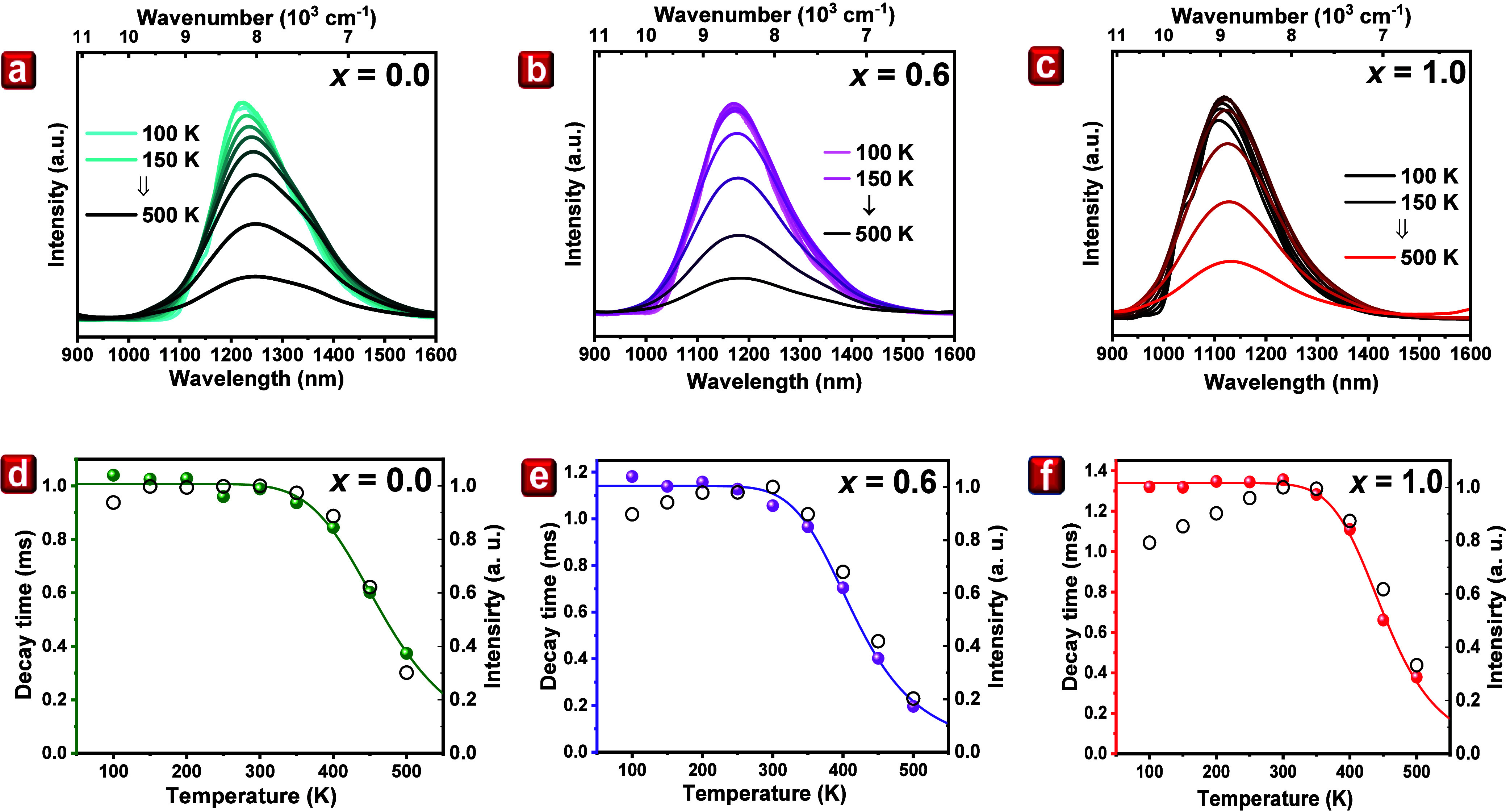
(a–c) Temperature-dependent photoluminescence spectra of Ni^2+^ emissions of LiGa_5(1–*x*)_Al_5*x*_O_8_:Cr^3+^,Ni^2+^ for *x* = 0.0, 0.6, and 1.0, respectively, under 405 nm excitation. (d–f) Calculated average decay times (full dots) and the integrated emission intensity (black circles) of LiGa_5(1–*x*)_Al_5*x*_O_8_:0.05Cr^3+^,Ni^2+^.

Panels a–c of Figure 4 illustrate the temperature-dependent photoluminescence (TDPL) spectra of Ni^2+^ across the temperature range of 100–500 K for samples with *x* values of 0.0, 0.6, and 1.0, respectively. All of the samples have a similar trend, where the emission intensity remains stable up to 300 K, followed by a rapid decrease. However, in the case of *x* = 0.6 and *x* = 1.0, luminescence increases below 300 K, which is not present in the *x* = 0.0 sample. It is important to note that, under an excitation wavelength of 405 nm, Ni^2+^ ions are indirectly excited through energy transfer from Cr^3+^ ions. Figure S6b provides a detailed explanation of this energy transfer mechanism. The process starts from the lowest excited ^2^E state of Cr^3+^ to the ^3^T_1_ excited state of Ni^2+^ ions, followed by a nonradiative transition to a lower ^3^T_2_ state from where luminescence is observed. The increased emission intensity at lower temperatures in the *x* = 0.6 and *x* = 1.0 samples can be explained by the positioning of the ^3^T_1_ level above the ^2^E level of Cr^3+^ ions. As the temperature increases, electrons can be in higher vibrational states of the ^2^E level, and the energy adjustment allows for the transfer to the ^3^T_1_ state of Ni^2+^. However, in the *x* = 0.0 sample, the energy levels align exactly or slightly below the ^2^E state of Cr^3+^ ions, eliminating the need for additional thermal energy to facilitate energy transfer. In summary, the increase of the PL intensity for the samples *x* = 0.6 and *x* = 1.0 is due to the positioning of the ^3^T_1_ level above the ^2^E level of the Cr^3+^. With the increase in temperature, the electrons can occupy higher vibrational levels of ^2^E, which allow for a more efficient energy transfer to the ^3^T_1_ level of Ni^2+^.

Furthermore, panels a–c of Figure S8 display the temperature-dependent decay profiles of Ni^2+^ emission for samples with *x* concentrations of 0.0, 0.6, and 1.0, respectively, ranging from 100 to 500 K. These decay profiles were recorded under direct Ni^2+^ excitation at 900 nm. It is noteworthy that all decay profiles exhibit multiexponential characteristics. Moreover, panels d–f of Figure 4 illustrate the calculated decay times as a function of temperature, represented by colored dots. There is a clear correlation between the temperature-dependent behavior of decay times and emission intensity. Notably, both decay time and emission intensity exhibit a pronounced decrease above 300 K, attributed to the nonradiative quenching of Ni^2+^. The temperature dependency of Ni^2+^ decay time was further analyzed using formula 2. The fitting curve is shown as a solid line, indicating the results of this analysis. The parameters derived from this fitting process, including the activation energy (*E*_A_), nonradiative parameter (*p*_nr_), and initial decay time (τ_0_), are summarized in Table 1.
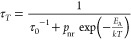
2The ^3^T_2_ → ^3^A_2_ transition probability is likely affected by the ^1^E state of Ni^2+^. In this situation, the ^1^E state interacts with the ^3^T_2_ state due to the quantum-mechanical mixing caused by spin–orbit interaction. The *x* = 1.0 sample has the longest decay time, caused by the influence of the closely lying ^1^E to the ^3^T_2_ state in the case of the strongest crystal field.

**Table 1:** Obtained Values of Fitting Parameters: *E*_A_, *p*_nr_, and τ_0_ for Ni^2+^ Luminescence

	*E*_A_(cm^–1^)	τ_0_ (ms)	ρ_nr_ (ms^–1^)
*X* = 0.0	2850 ± 350	1.01 ± 0.01	(6 ± 6) × 10^3^
*X* = 0.6	2600 ± 220	1.14 ± 0.01	(6 ± 5) × 10^3^
*X* = 1.0	3400 ± 250	1.34 ± 0.01	(4 ± 3) × 10^4^

**Figure 5:**
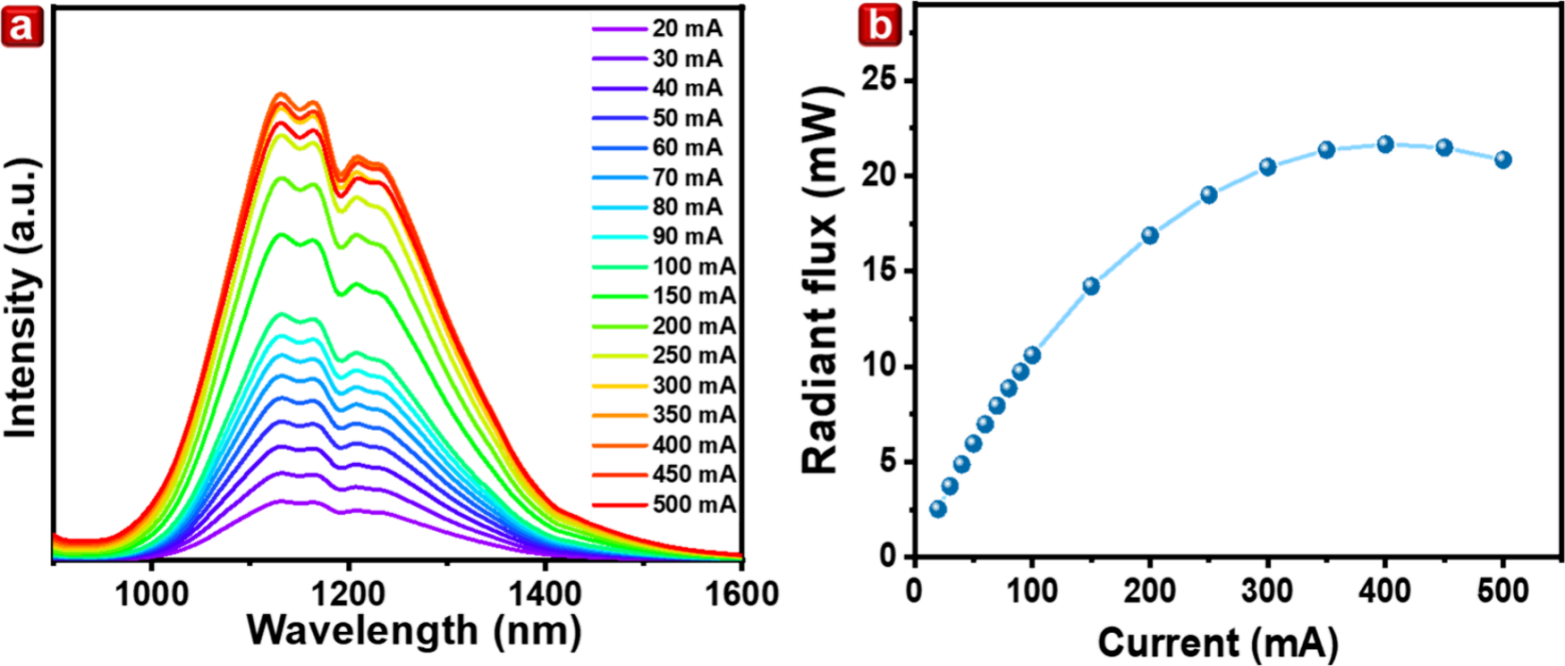
(a) Current-dependent spectra of SWIR LED by LiGa_5(1–*x*)_Al_5*x*_O_8_:0.05Cr^3+^,0.015Ni^2+^ (*x* = 0.6) phosphor. (b) Radiant flux under different currents.

As indicated in the Introduction, SWIR fluorescence imaging has become a pivotal tool in digital medical applications, agriculture, and semiconductor technology.^30−33^ SWIR light has a significant advantage in biological contexts due to its ability to penetrate biotissue with minimal photon absorption and scattering.^34^ This property is primarily due to the reduced energy loss during light propagation, which can be attributed to absorption decay and scattering disturbances.^35^ This makes SWIR light particularly suitable for bioimaging applications, offering high-quality images with minimal interference. In this study, a SWIR-LED is constructed utilizing LGAOCN with *x* = 0.6 phosphors integrated onto 450 nm blue LED (double chip of 26 × 46 mil) on a CREE XP1-LED board, providing less water absorption in 1380–1400 nm compared to the device using *x* = 0.0 sample. The resin ratio between phosphor and silicone gel is approximately 1:1. Figure 5a illustrates the spectral radiation flux under various driving currents, showcasing the high efficiency of SWIR-LED. Surprisingly, the spectrum reveals distinctive characteristic absorptions at 1150, 1190, and 1224 nm. This observation is attributed to molecular vibrations in the silicon package gel, where fundamental absorptions occur ranging from the first to the third overtone, per the harmonic approximation. These absorption dips are particularly noticeable in hydrogen-based bonds such as C–H, O–H, and N–H.^36^ These bonds exhibit higher vibrational energies in the SWIR region, encompassing stretching and bending modes. Specifically, the second overtones of functional groups such as CH_3_–, CH_2_–, and CH– exhibit absorption in the 1100–1200 nm range, and ArOH exhibits absorption in the 1200–1300 nm range. The absorption characteristic of a substance is crucial in identifying its functional groups. This aids in distinguishing between different components. Nevertheless, a notable phenomenon arises wherein the silicon package gel, commonly used in LED devices, may absorb emissions from SWIR phosphors.

The saturation flux, depicted in Figure 5b, reaches a high-power flux of approximately 21.45 mW when driven by a current of 400 mA. Subsequently, a decrease in radiant flux is observed, which can be attributed to the thermal quenching of the phosphor. Panels a–d of Figure S9 reveal finger and palm images captured using transmission and reflection methods. In this case, the SWIR image was captured by a digital VIS-SWIR camera (Alizé 1.7) with a 900 nm long pass filter. In comparison to previously studied NIR images (with a 600 nm filter), SWIR images also reveal vascular tissues, signifying their applicability in biological imaging.^15,22^ However, SWIR can induce molecular vibrational absorption compared to NIR LEDs, affecting tissue penetration. For example, the light in the SWIR region is absorbed by water at 1400 nm, whereas the light in the NIR region is absorbed by melanin and hemoglobin at 800–1000 nm. Therefore, NIR and SWIR each have their respective advantages in biological applications. By tuning different wavelengths, it is feasible to target appropriate tissue imaging applications.

## 3 Conclusion

In conclusion, a series of LiGa_5(1–*x*)_Al_5*x*_O_8_:0.05Cr^3+^,0.015Ni^2+^ (*x* = 0.0–1.0) phosphors were synthesized by solid-state reaction. XRD analysis revealed peak shifts to higher angles due to Al doping, accompanied by a distortion angle increase of GaO_6_ and AlO_6_ from 90° (*x* = 0.0) to 98° (*x* = 1.0). Raman spectroscopy showed a decrease in symmetry due to Al doping, resulting in reduced Raman activity. XANES results highlighted lattice contraction and enhanced ionic bonding of Cr^3+^, resulting in a reduced effective shielding of the nuclear charge; however, Ni^2+^ showed the opposite result. Cr^3+^ and Ni^2+^ have different coordination environments, proved by the X-ray absorption spectra, showing the possibility that they occupy different positions or that the coordinated environment of Ni^2+^ is distorted due to charge imbalance. For optical properties, the energy transfer from Cr^3+^ (sharp emission) to Ni^2+^ (broad emission) emitted in the 1000–1600 nm range was observed. When *x* increases, the energy transfer from ^2^E (Cr^3+^) to ^3^T_1_ (Ni^2+^) efficiency decreases and becomes temperature-dependent due to the energy level mismatch. Temperature-dependent photoluminescence and decay profiles were used to infer Ni crystal field change trends. Finally, high-power SWIR LED packaging measured a radiant flux of up to 21.45 mW, confirming its applicability in biological imaging. This research extensively contributes to understanding dual-site replacement and energy transfer analysis in SWIR phosphor.

## 4 Experimental Method

This study used high-temperature solid-state synthesis to prepare samples of LiGa_5(1–*x*)_Al_5*x*_O_8_:0.05Cr^3+^,0.015Ni^2+^ (*x* = 0.0–1.0). First, the precursors such as Li_2_CO_3_ (99.99%, Thermo Fisher Scientific), Ga_2_O_3_ (99.99%, Gredmann), NiO (99.99%, LTS (Chemical) Inc.), Cr_2_O_3_ (99.99%, Gredmann), and Al_2_O_3_ (99.99%, Gredmann) was ground by mortar with molar ratio. The powder mixture was ground in a mortar until uniformly fine and placed in an alumina crucible. The sample was then loaded into a tube furnace and heated to 800 °C (2 h) and 1300 °C (4 h) in air at a 5 °C/min rate. After sintering, the sample was allowed to cool to room temperature and ground again into powder for subsequent analyses.

## Supporting Information Available

Characterization; X-ray diffractions; Rietveld refinement; crystal parameters; scanning electron microscopy; optical microscopy; temperature-dependent decay profile; short-wave infrared application photograph (PDF)
