# Supplementary material for: Sharp-to-Broad Band Energy Transfer in Lithium Aluminate and Gallate Phosphors for SWIR LED
Source: ACS Appl Opt Mater. 2024 Mar 1;2(12):2401–8. doi: 10.1021/acsaom.3c00464 (PMC11686514; doi:10.1021/acsaom.3c00464)
Supplement: Supplementary file 1 — ot3c00464_si_001.pdf [file ot3c00464_si_001.pdf]

Supporting Information

Sharp-to-Broad Band Energy Transfer in Lithium Aluminate and Gallate Phosphors for SWIR LED

Yi-Ting Tsai,[†] Pei-Xuan Chen,[†,§] Mikołaj Kamiński,[Ψ] Natalia Majewska,[Ψ] Sebastian Mahlik,[Ψ,‡,*] and Mu-Huai Fang[†,*]

[†]Research Center for Applied Sciences, Academia Sinica, Taipei 11529, Taiwan

[§]Institute of Organic and Polymeric Materials, National Taipei University of Technology, Taipei 10608, Taiwan

[Ψ]Institute of Experimental Physics, Faculty of Mathematics, Physics and Informatics, University of Gdansk, Wita Stwosza 57, 80-308 Gdansk, Poland

[‡]International Centre for Theory of Quantum Technologies (ICTQT), University of Gdansk, 80-308 Gdansk, Poland

Correspondence to: *fangmuhuai@gate.sinica.edu.tw (M.H.F.), and *sebastian.mahlik@ug.edu.pl (S.M.)

Characterization

Synchrotron Powder X-ray diffraction patterns were analyzed at the National Synchrotron Radiation Research Center (NSRRC) in Taiwan, utilizing the BL01C2 beamline. These analyses were conducted at room temperature (RT). Total Pattern Analysis Solutions software (TOPAS 6.0) was used for Rietveld refinement of the

synchrotron diffraction patterns. Additionally, the X-ray absorption characteristics of Cr and Ni were investigated using both the Cr *K*-edge and Ni *K*-edge X-ray absorption near-edge structure (XANES) and extended X-ray absorption fine structure (EXAFS) techniques in the BL44A1 beamline. RT photoluminescence excitation (PLE) spectra of visible (red) emission of Cr^{3+} were measured with a FluoroMax-4P spectrofluorometer (Horiba) equipped with a 150 W xenon lamp as an excitation source and an R928 Hamamatsu photomultiplier as a detector which allows recording photoluminescence (PL) and PLE spectra in the spectral range of 250–850 nm. RT PLE spectra of near-infrared emission of Ni^{2+} were measured using a custom-made setup consisting of EQ99X laser-driven Xe light source (Energetiq) coupled to a self-made grating monochromator operating between 250–1000 nm as the excitation source and the Andor SR-500i-D1 spectrometer equipped with a CCD camera (DU490A-1.7) operating in the 600–1700 nm wavelength as luminescence detector. The temperature-dependent Cr^{3+} PL spectra were measured using an Andor SR-750–D1 spectrometer equipped with a CCD camera (DU420A-OE) operating in the 350–1000 nm wavelength. The temperature and pressure-dependent NIR PL spectra were measured using an Andor SR-500i-D1 spectrometer equipped with a CCD camera (DU490A-1.7) operating in the 600–1700 nm wavelength. A light-emitting diode with maximum emission at 410 nm and the DPSS laser with 473 nm emission were used as excitation sources for temperature-dependent and pressure-dependent measurements, respectively. The temperature-dependent decay profiles were measured using a custom-made setup consisting of a function generator to generate the square-shaped light pulses of duration 100 ms and fall time ca. 100 ns, the National Instrument Data Acquisition module, and Avalanche Photodetector APD110A2/M operated in the spectral range 200–1000 nm, and APD110C/M operated in 900–1700 nm for Cr^{3+} and Ni^{2+}, respectively. Spectroscopic studies as a function of temperature were obtained using

the THMS600 Linkam stage temperature controller combined with the LNP95 liquid nitrogen cooling pump system, allowing the temperature to be obtained in the range of 100–600 K. Diffuse reflectance (DR) spectra were measured using a Hamamatsu Absolute PL quantum yield spectrometer C11347 series.

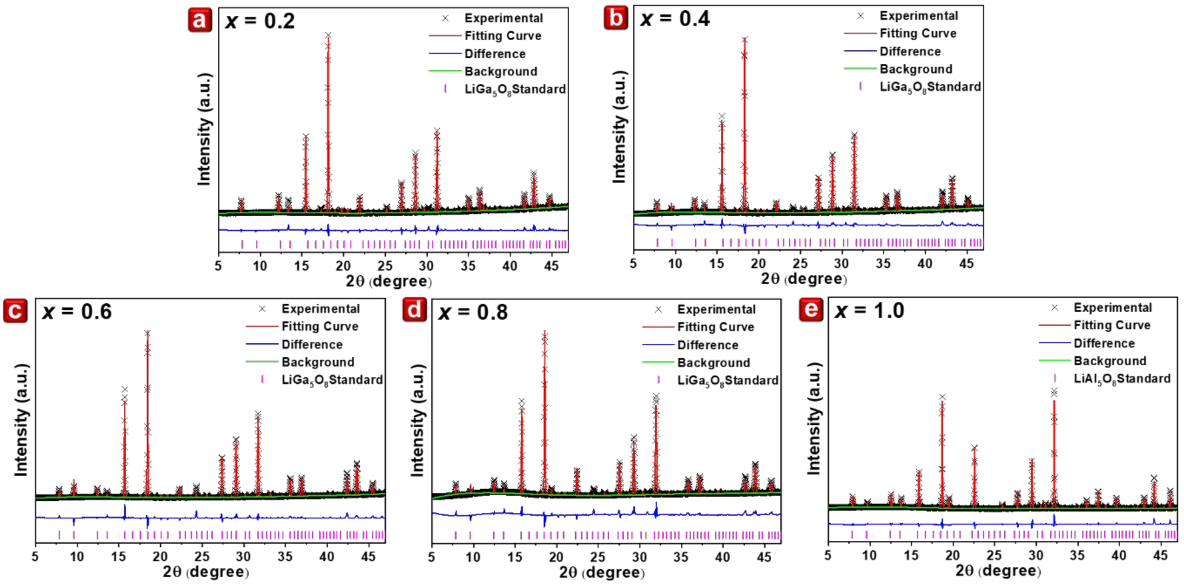

Figure S1. XRD refinement of $LiGa_{5(1-x)}Al_{5x}O_8$:$0.05Cr^{3+}$,$0.015Ni^{2+}$ for x = 0.2–1.0. Although the XRD CIF peak positions of $LiGa_5O_8$ and $LiAl_5O_8$ are close, using $LiAl_5O_8$ yields better refinement results due to local distortion at x = 1.0.

Table S1. Atomic positions, occupancies, and atomic displacement parameters of $LiGa_{5(1-x)}Al_{5x}O_8$:$0.05Cr^{3+}$,$0.015Ni^{2+}$ for x = 0.0–1.0.

x = 0.0						
Site	Np	*x*	*y*	*z*	*Occ*	*Beq*
Li1	4	0.625	0.625	0.625	1	1.7(12)
Ga1	12	0.125	0.375	0.875	1	0.18(3)
Ga2	8	0	0	0	1	0.18(3)
O1	8	0.375	0.375	0.375	1	0.38(10)
O2	24	0.125	0.125	0.375	1	0.38(10)

x = 0.2						
Site	Np	*x*	*y*	*z*	*Occ*	*Beq*
Li1	4	0.625	0.625	0.625	1	0.5(9)
Ga1	12	0.125	0.375	0.875	0.678(2)	0.49(4)
Al1	12	0.125	0.375	0.875	0.322(2)	0.49(4)
Ga2	8	0	0	0	0.983(3)	0.49(4)
Al2	8	0	0	0	0.017(3)	0.49(4)
O1	8	0.375	0.375	0.375	1	0.94(9)
O2	24	0.125	0.125	0.375	1	0.94(9)

x = 0.4						
Site	Np	*x*	*y*	*z*	*Occ*	*Beq*
Li1	4	0.625	0.625	0.625	1	2.4(11)
Ga1	12	0.125	0.375	0.875	0.389(19)	0.29(4)
Al1	12	0.125	0.375	0.875	0.611(19)	0.29(4)
Ga2	8	0	0	0	0.917(3)	0.29(4)
Al2	8	0	0	0	0.083(3)	0.29(4)
O1	8	0.375	0.375	0.375	1	1.22(10)
O2	24	0.125	0.125	0.375	1	1.22(10)

x = 0.6						
Site	Np	*x*	*y*	*z*	*Occ*	*Beq*
Li1	4	0.625	0.625	0.625	1	5.9(19)
Ga1	12	0.125	0.375	0.875	0.156(2)	0.32(4)
Al1	12	0.125	0.375	0.875	0.844(2)	0.32(4)
Ga2	8	0	0	0	0.766(3)	0.32(4)
Al2	8	0	0	0	0.234(3)	0.32(4)
O1	8	0.375	0.375	0.375	1	1.44(12)
O2	24	0.125	0.125	0.375	1	1.44(12)

x = 0.8						
Site	Np	*x*	*y*	*z*	*Occ*	*Beq*
Li1	4	0.625	0.625	0.625	1	4.3(16)
Ga1	12	0.125	0.375	0.875	0.060(2)	0.22(6)
Al1	12	0.125	0.375	0.875	0.940(2)	0.22(6)
Ga2	8	0	0	0	0.410(3)	0.22(6)
Al2	8	0	0	0	0.590(3)	0.22(6)
O1	8	0.375	0.375	0.375	1	1.04(11)
O2	24	0.125	0.125	0.375	1	1.04(11)

x = 1.0						
Site	Np	*x*	*y*	*z*	*Occ*	*Beq*
O1	24	0.1159(8)	0.1335(5)	0.383(5)	1	1
O2	8	0.3851(4)	0.3851(4)	0.385(4)	1	1
Al1	8	0.9988(2)	0.9988(2)	0.998(2)	1	1
Al2	4	0.625	0.625	0.625	0.043	1
Li1	4	0.625	0.625	0.625	0.957	1
Al3	12	0.3686	0.8814	0.125	0.986	1
Li2	12	0.3686	0.8814	0.125	0.014	1

Table S2. Refined parameters of $LiGa_{5(1-x)}Al_{5x}O_8$:$0.05Cr^{3+}$,$0015Ni^{2+}$ for x = 0.0–1.0.

x	0.0	0.2	0.4	0.6	0.8	1.0 ($LiAl_5O_8$ CIF)
a (Å)	8.20348(7)	8.14554(13)	8.07993(15)	8.01832(16)	7.9622(3)	7.9106(2)
V (Å³)	552.071(14)	540.45(3)	527.50(3)	515.52(3)	504.77(5)	495.02(4)
R_{wp} (%)	4.33	3.45	3.97	6.45	6.81	7.79
R_p (%)	2.28	2.10	2.51	3.94	4.93	6.19

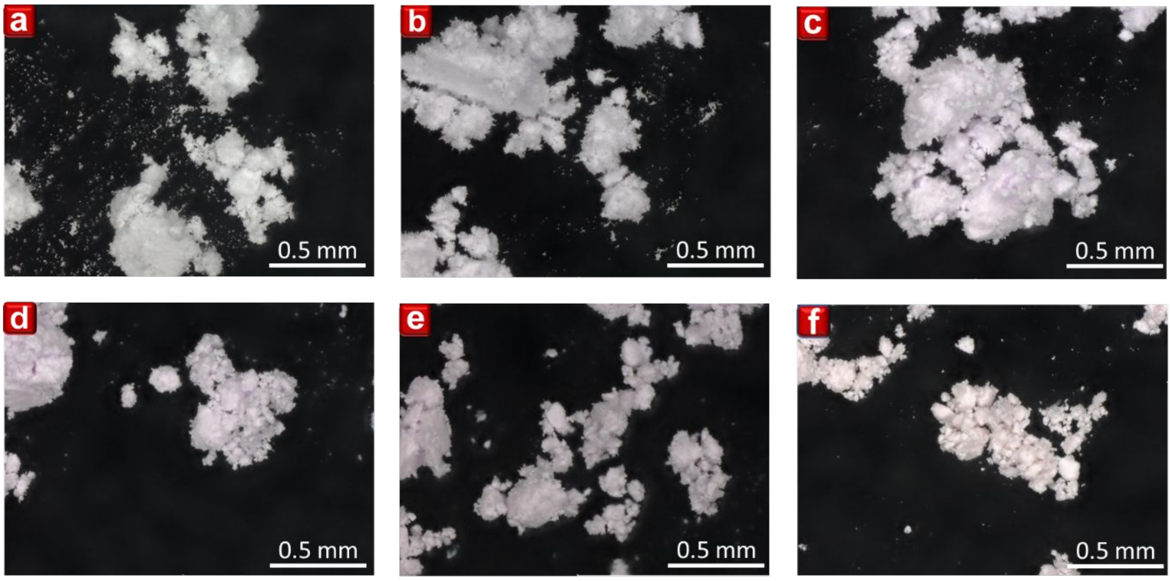

Figure S2. Optical microscopy images of $LiGa_{5(1-x)}Al_{5x}O_8$:$0.05Cr^{3+}$,$0.015Ni^{2+}$ for (a) $x = 0.0$, (b) $x = 0.2$, (c) $x = 0.4$, (d) $x = 0.6$, (e) $x = 0.8$, and (f) $x = 1.0$.

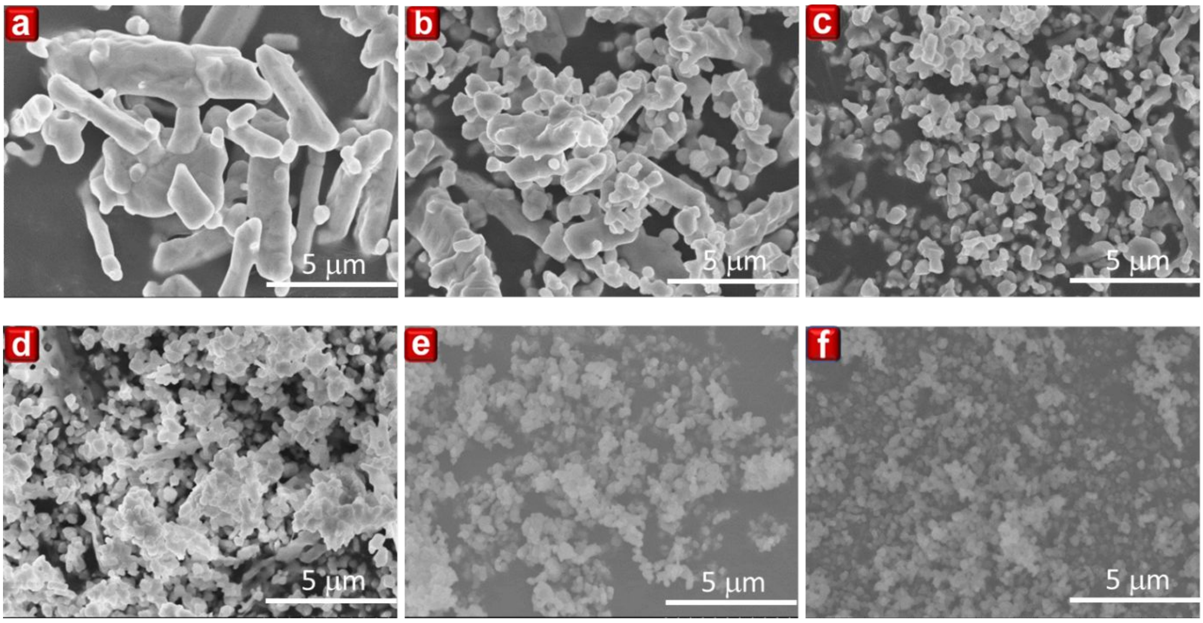

Figure S3. Scanning electron microscopy images of $LiGa_{5(1-x)}Al_{5x}O_8$:0.05Cr^{3+},0.015Ni^{2+} for (a) x = 0.0, (b) x = 0.2, (c) x = 0.4, (d) x = 0.6, (e) x = 0.8, and (f) x = 1.0.

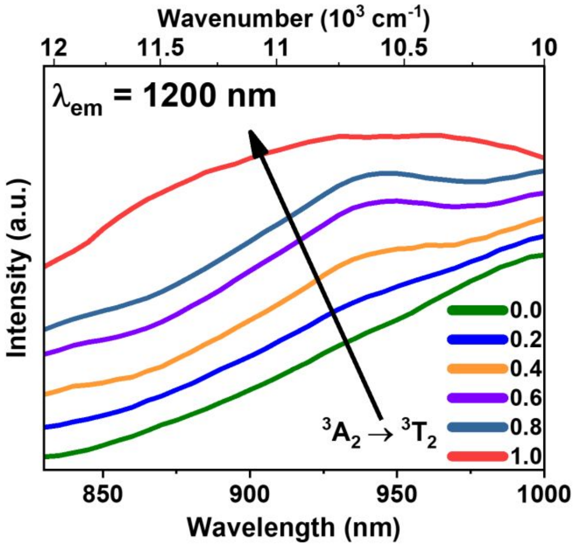

Figure S4. The position of the $^3A_2 \rightarrow {}^3T_2$ transition band of Ni^{2+} excitation spectra of $LiGa_{5(1-x)}Al_{5x}O_8$:0.05Cr^{3+},0.015Ni^{2+} with x = 0.0–1.0.

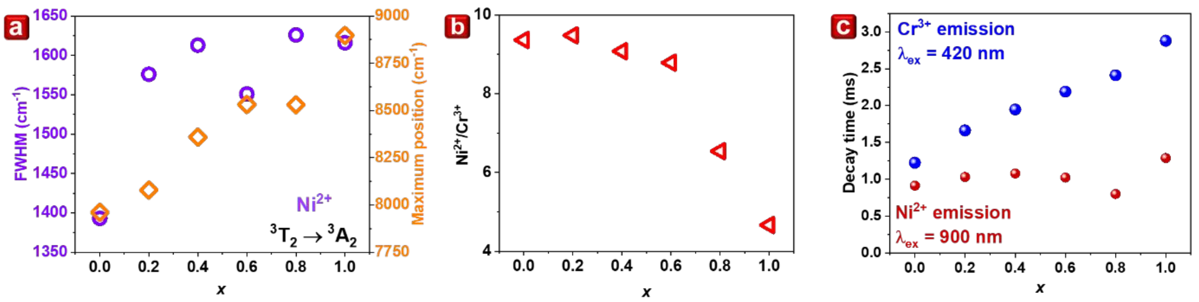

Figure S5. (a) The full width at half maximum (FWHM) value and the position of the Ni^{2+} emission maximum. (b) The Ni^{2+}/Cr^{3+} ratio. (c) Average decay time for Cr^{3+} (excitation at 420 nm, observation at 700–740 nm) and Ni^{2+} (excitation at 900 nm, observation at 1000–1400 nm) emission of the $LiGa_{5(1-x)}Al_{5x}O_8$:0.05Cr^{3+},0.015Ni^{2+} with x = 0.0–1.0.

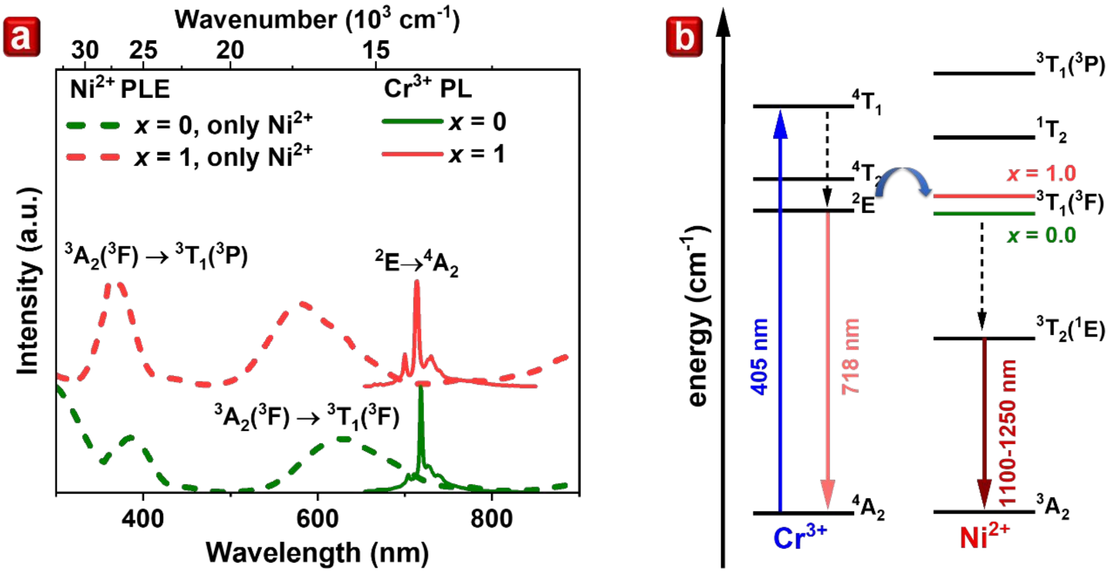

Figure S6. Evidence of the energy transfer mechanism. (a) Photoluminescence excitation spectra of $LiGa_{5(1-x)}Al_{5x}O_8$ doped only with Ni^{2+} and photoluminescence spectra of Cr^{3+} ions, revealing the Cr^{3+} emission spectra and Ni^{2+} excitation spectra overlap. (b) Scheme of the energy transfer from Cr^{3+} to Ni^{2+} and crystal field effect on $^3T_1(^3F)$ state of $LiGa_{5(1-x)}Al_{5x}O_8$:0.05Cr^{3+},0.015Ni^{2+}.

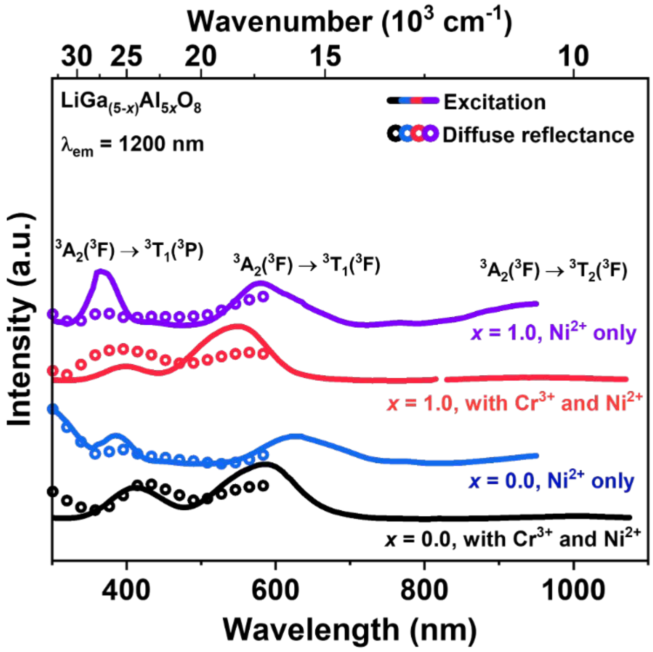

Figure S7. The photoluminescence excitation spectra (solid lines) of the Ni^{2+} emission and diffuse reflectance spectra (circles) of $LiGa_{5(1-x)}Al_{5x}O_8$ doped with Ni and Cr, and only with Ni for x = 0.0 and 1.0.

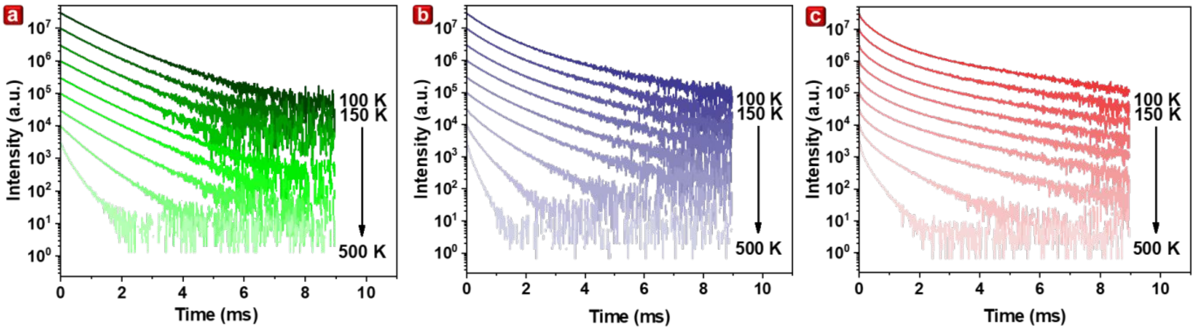

Figure S8. Temperature-dependent decay profiles of Ni^{2+} emission (a) x = 0.0, (b) 0.6, and (c) 1.0 under 900 nm excitation (direct Ni^{2+} excitation of $LiGa_{5(1-x)}Al_{5x}O_8$:$0.05Cr^{3+}$,$0.015Ni^{2+}$) and observation at 1000–1400 nm.

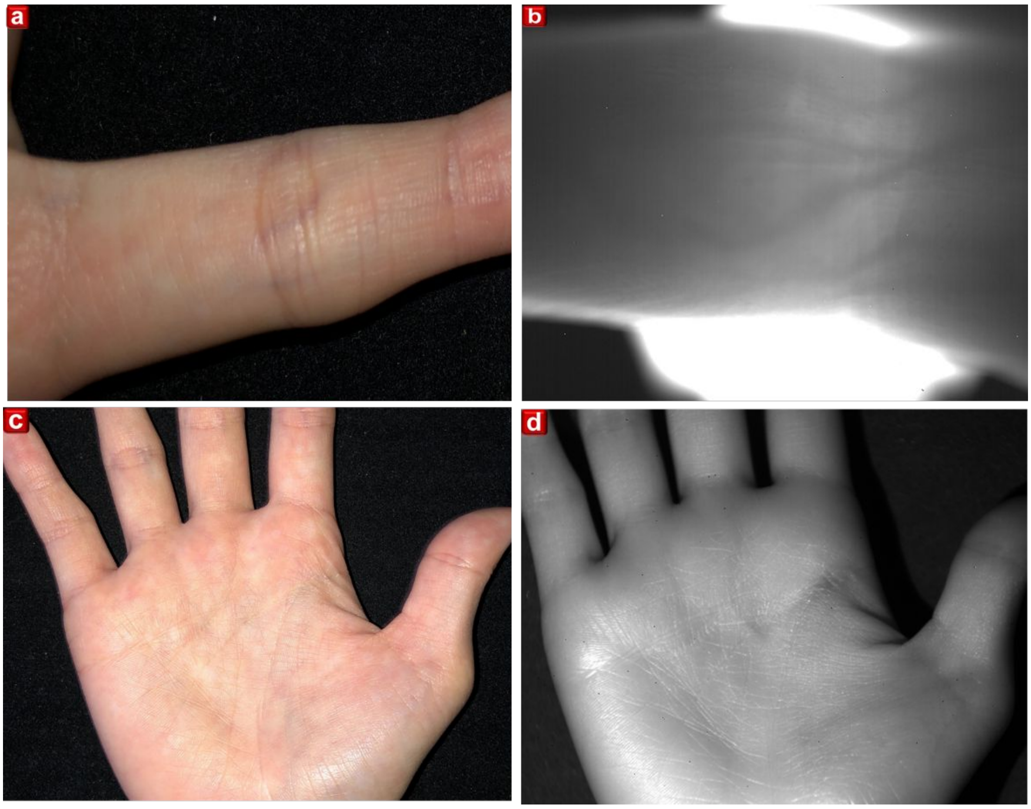

Figure S9. (a,b) Visible light and SWIR photo of a finger using transmission method. (c,d) Visible light and SWIR photo of the palm by using the reflection method.
